# Mdivi-1-Sensitive Mitochondrial Remodeling Contributes to B Cell Immune Synapse Formation and Antigen Presentation

**DOI:** 10.3390/cells15121114

**Published:** 2026-06-19

**Authors:** Juan Pablo Bozo, Teemly Contreras, Antonio Sánchez-Squella, Jheimmy Diaz-Muñoz, María-Isabel Yuseff

**Affiliations:** 1 Laboratory of Immune Cell Biology, Faculty of Biological Sciences, Pontificia Universidad Católica de Chile, Santiago 8331150, Chile; jpbozo@uc.cl (J.P.B.); tvcontreras@uc.cl (T.C.); jdiazm@uc.cl (J.D.-M.); 2 Department of Electrical Engineering, Universidad Técnica Federico Santa María, Santiago 8320000, Chile; antonio.sanchez@usm.cl

**Keywords:** Drp1, mitochondria, B cell immune synapse

## Abstract

B cell activation requires the formation of an immune synapse (IS), where coordinated cytoskeletal remodeling and organelle dynamics enable antigen extraction and presentation. While mitochondria are known to regulate cellular metabolism during activation, their role in IS function remains poorly understood. Here, we investigated how mitochondrial dynamics influence antigen processing and presentation in B cells. We show that B cell receptor (BCR) engagement induces rapid phosphorylation of the mitochondrial fission GTPase Drp1 at Ser616. Treatment with mdivi-1, a compound used to perturb Drp1-associated mitochondrial fission that can also affect mitochondrial complex I activity, altered mitochondrial morphology, reduced mitochondrial activity, and decreased their stable accumulation at the synapse. This was accompanied by increased tubulin acetylation, lysosome retention near the MTOC, and reduced delivery to the synaptic membrane. Accordingly, lysosome fusion, antigen extraction, and presentation to T cells were significantly diminished in mdivi-1-treated B cells. Together, our findings suggest that mdivi-1-sensitive mitochondrial fission and activity are associated with mitochondrial positioning, lysosomal trafficking, and exocytosis at the B cell immune synapse, supporting a model in which mitochondrial dynamics contribute to efficient antigen extraction and presentation.

## 1. Introduction

Antigen recognition by the B cell receptor (BCR) triggers the formation of an immune synapse (IS), where BCR signaling and antigen extraction are tightly regulated [1,2]. The formation of a functional immune synapse requires coordinated remodeling of the actin cytoskeleton and microtubule network, which together direct the polarized trafficking and positioning of lysosomes at the synaptic membrane [3,4,5]. At the synapse, dynein-dependent lysosome transport enables lysosome docking and fusion with the plasma membrane, leading to the local release of acidic contents that facilitate antigen extraction [1,6]. Extracted antigens are subsequently internalized into endolysosomal compartments, where they are processed and loaded onto MHC-II molecules for presentation to CD4+ T lymphocytes [1,6].

Because immune synapse formation depends on polarized organelle trafficking and positioning, the mechanisms that coordinate these events are central to efficient antigen extraction and presentation [3,7]. While the roles of the actin and microtubule cytoskeletons in this process have been extensively studied, the contribution of mitochondria to the spatial regulation of the B cell synaptic interface remains poorly understood.

Mitochondria are key regulators of B cell metabolism, supporting the bioenergetic demands of activation and shaping the metabolic program that follows antigen encounter [8,9,10]. Beyond ATP production, mitochondria buffer cytosolic Ca^2+^ and thereby contribute to the tuning of BCR signaling [11,12,13]. More broadly, mitochondrial metabolism is closely linked to immune cell effector functions, including activation, differentiation, and survival [8,9,10,11,12,13,14,15]. These functions depend not only on mitochondrial bioenergetics, but also on mitochondrial dynamics, including directed transport along microtubules and cycles of fusion and fission that remodel organelle architecture [14,15]. Mitochondrial fission is executed by the cytosolic GTPase Drp1, which is recruited to the outer mitochondrial membrane by receptors including Fis1 and the MiD proteins (MiD49/MIEF1 and MiD51/MIEF2), and is closely linked to metabolic control [16,17,18].

Evidence from T cells supports the idea that mitochondrial positioning and dynamics can directly regulate immune synapse-associated functions. In T cells, mitochondria relocalize toward the immune synapse, where they contribute to local Ca^2+^ buffering and sustain T cell receptor signaling during activation [19]. In addition, Drp1-dependent mitochondrial fission has been shown to regulate mitochondrial redistribution and modulate T cell receptor signaling at the immune synapse [20]. Together, these studies indicate that mitochondria act not only as metabolic organelles, but also as spatially organized regulators of lymphocyte synapse function.

In B cells, mitochondria have also recently emerged as regulators of processes linked to antigen presentation. In a B cell-specific Tfam loss-of-function model, defective mitochondrial remodeling impaired lysosomal compartment reorganization, disrupted antigen presentation, and compromised germinal center formation, ultimately promoting the features of an aged humoral response. Notably, these defects were not primarily attributable to reduced bioenergetic supply, suggesting that mitochondria influence B cell function through signaling pathways that extend beyond ATP production [21].

Despite these observations, the role of mitochondrial dynamics during B cell immune synapse formation, and its impact on antigen extraction and presentation, remain unknown. Here, we examined how mitochondrial dynamics are regulated when B cells engage immobilized antigens that promote immune synapse formation, and whether these dynamics contribute to antigen extraction, processing, and presentation. We find that BCR engagement rapidly induces phosphorylation of Drp1, the GTPase that drives mitochondrial fission. Treatment with mdivi-1, used here as a pharmacological perturbation of Drp1-associated mitochondrial remodeling and mitochondrial activity, was associated with reduced mitochondrial accumulation at the B cell synapse, decreased lysosome docking and fusion at the plasma membrane, and impaired antigen extraction and presentation. Thus, mdivi-1-sensitive mitochondrial remodeling emerges as a candidate process linked to efficient antigen capture and presentation to T cells.

## 2. Materials and Methods

### 2.1. Cell Culture

The IIA1.6 cell line corresponds to mouse B-cell lymphoma defective in FcγR, displaying the phenotype of quiescent mature B lymphocytes, and was employed in main experiments [22]. In contrast, the LMR7.5 cell line originates from a T-cell hybridoma specific to the LACK antigen, is capable of recognizing I-Ad-LACK156–173 complexes, and was used in presentation assays. Both cell lines were cultured under previously described conditions [4], in CLICK medium (RPMI 1640 supplemented with 10% fetal bovine serum, 100 U/mL penicillin-streptomycin, 0.1% β-mercaptoethanol, and 2% sodium pyruvate), within a cell culture incubator at 37 °C and 5% CO_2_.

### 2.2. Reagents and Antibodies

For immunofluorescence, the following primary antibodies were employed: F(ab’)_2_ goat anti-mouse immunoglobulin G (IgG) (Jackson ImmunoResearch, West Grove, PA, USA); rabbit anti-DNM1L (Drp1) (Invitrogen, Eugene, OR, USA; PA1-16987, 1:300); rabbit anti-acetylated α-tubulin (Cell Signaling Technology, Danvers, MA, USA; #5335T, 1:200); rat anti-LAMP1/CD107a (BD Biosciences, Franklin Lakes, NJ, USA; #553792, 1:200); rabbit anti-OVA (Sigma-Aldrich, Burlington, MA, USA; #C6534, 1:500); rabbit anti-TOM20 (Cell Signaling Technology, #42406, 1:300); and rabbit anti-phospho-DRP1 (Ser616, pDrp1Ser616) (Cell Signaling Technology, Danvers, MA, USA; #3455, 1:300).

Secondary antibodies included: Alexa Fluor 488-conjugated goat anti-rabbit (Life Technologies, Carlsbad, CA, USA, 1:200); Alexa Fluor 647 and Cy3-conjugated F(ab’)_2_ donkey anti-rat, as well as Cy3–conjugated F(ab’)_2_ donkey anti-rabbit (Jackson ImmunoResearch, 1:200); Rhodamine Phalloidin (Invitrogen #R415, 1:200); Alexa Fluor 488 F(ab’)_2_ donkey anti-chicken IgG (H + L) (Jackson ImmunoResearch, West Grove, PA, USA; #703-546-155, 1:300); and F(ab’)_2_ Alexa Fluor 546–conjugated goat anti-mouse IgG together with Alexa Fluor 647-conjugated F(ab’)_2_ goat anti-mouse IgG (Invitrogen, #A11018). Additionally, Alexa Fluor 488-conjugated F(ab’)_2_ goat anti-mouse IgG (Jackson ImmunoResearch, Eugene, OR, USA) was used.

For Western blotting, the antibodies used were: rabbit anti-DNM1L (Drp1) (Invitrogen PA1-16987, 1:1000); rabbit anti-acetylated α-tubulin (Cell Signaling Technology, Eugene, OR, USA; #5335T, 1:1000); rabbit anti-phospho-DRP1 (Ser616, pDrp1Ser616) (Cell Signaling Technology, Danvers, MA, USA; #3455, 1:1000); GAPDH (Sigma-Aldrich, #G9545, Burlington, MA, USA; 1:1000); and ERK/pERK (#4695s and #9101s, respectively, 1:1000). Secondary detection was performed using HRP-conjugated donkey anti-rat, anti-rabbit, or anti-mouse antibodies (Jackson ImmunoResearch, West Grove, PA, USA; 1:5000).

CypHer 5E was obtained from Amersham Biosciences, Little Chalfont, Buckinghamshire, UK and used according to the manufacturer’s instructions. For mitochondrial disruption assays, cells were treated with 50 µM mdivi-1 (Sigma-Aldrich, #338967-87-6).

### 2.3. Western Blot

Cells were lysed using 40 µL of RIPA buffer, after which the lysates were collected, resolved by gel electrophoresis, and transferred onto polyvinylidene fluoride membranes (Trans-Blot Semi-Dry Transfer Cell; Bio-Rad, Hercules, CA, USA). Membranes were blocked with 2% BSA in TBS containing 0.05% Tween-20 and subsequently incubated overnight at 4 °C with primary antibodies, followed by a 60 min incubation with secondary antibodies. Western blots were developed using the Westar Supernova substrate (Cyanagen, Bologna, Italy Cat. No. XLS-3,0100), and chemiluminescent signals were detected with the iBright imaging system (Thermo Fisher, Bothell, WA, USA).

### 2.4. Activation of B Cells Using Immobilized Antigens

#### 2.4.1. Preparation of BCR Ligand-Coated Beads

A total of 4 × 10^7^ 3 µm latex NH_2_ beads (Polysciences, Eppelheim, Germany) were activated with 8% glutaraldehyde (Sigma-Aldrich) for 4 h at room temperature [5,7,23]. Following activation, the beads were washed with PBS and subsequently incubated overnight at 4 °C with different ligands: either 100 µg/mL F(ab′)_2_ goat anti-mouse IgG (designated as BCR ligand + beads) or 100 µg/mL F(ab′)_2_ goat anti-mouse IgM (BCR ligand − beads; MP Biomedical, Santa Ana, CA, USA). These incubations were performed either alone or in combination with 100 µg/mL of the *Leishmania major* antigen LACK or 100 µg/mL of OVA protein.

#### 2.4.2. B Cell Stimulation and Immunofluorescence

Cells were seeded onto poly-L-lysine-coated slides and stimulated with the indicated beads at a 1:1 cell-to-bead ratio for the specified times at 37 °C. Cells were then fixed with 4% paraformaldehyde (PFA) for 10 min at room temperature. Following fixation, cells were blocked in PBS/BSA/Glycine (1×/2%/100 mM) for 10 min and subsequently incubated overnight with primary antibodies. After washing, cells were incubated for 1 h with secondary antibodies in PBS/BSA/Saponin (1×/0.2%/0.05%), rinsed with PBS, and mounted on slides using Fluoromount-G (Electron Microscopy Sciences, Hatfield, PA, USA). For experiments involving pharmacological treatment, B cells exposed to mdivi-1 or DMSO were pre-incubated with the respective reagents for 4 h prior to activation.

### 2.5. Presentation Assay

IIA1.6 B lymphocytes were treated with either DMSO or 50 µM mdivi-1 for 4 h, followed by activation with beads containing BCR+ ligand plus LACK (activated condition) or beads with non-BCR-related ligand plus LACK (non-activated condition). After stimulation, cells were washed with PBS, fixed in ice-cold PBS supplemented with 0.01% glutaraldehyde for 1 min, and quenched with PBS containing 100 mM glycine. Subsequently, B cells were co-cultured with LACK-specific T-cell hybridomas at a 1:1 ratio for 4 h [5]. Supernatants were collected, and IL-2 cytokine production was quantified using the BD OptEIA Mouse IL-2 ELISA kit, according to the manufacturer’s instructions (BD Biosciences).

### 2.6. Cell Imaging and Image Analysis

For widefield imaging, Z-stack acquisitions were performed with a step size of 0.3 µm between slices. Images were collected using a Nikon Ti Eclipse widefield epifluorescence microscope equipped with a 60×/1.25 NA objective (Nikon instrument, Melville, NY, USA). Confocal imaging was carried out on a Zeiss LSM880 microscope with Airyscan detection (Jena, Germany), employing a 63×/1.4 NA oil immersion lens and a Z-stack interval of 0.2 µm. All experimental replicates were acquired under identical illumination intensities (mW/cm^2^) or equivalent exposure conditions. Image processing was performed with Zeiss ZEN 2.3 Black software for Airyscan reconstruction, and subsequent analyses were conducted using Fiji (ImageJ 2.9). For colocalization studies, all planes from individual B cells were included and quantified using the JaCoP plugin in Fiji, following the developer’s instructions. The accumulation of lysosomes at the IS was quantified by measuring the LAMP-1 fluorescence intensity in a concentric circular area closely surrounding the bead (3.5 μm) and normalized by the whole-cell LAMP-1 fluorescence intensity [5]. Determination of the localization index for lysosomal distribution between the central and peripheral regions of the cell was adapted from previously published methods [23].

From confocal datasets, each cell was isolated from the field of view and reconstructed independently in three dimensions using ImageJ. Quantification of discrete clusters and their volumes was performed with the ImageJ 3D Object Counter plugin.

### 2.7. Statistical Analysis

Data are expressed as mean ± SEM, and differences were considered statistically significant at *p* < 0.05. Statistical analyses were performed using Student’s *t*-test when comparing two experimental groups, one-way ANOVA for datasets with three or more groups involving a single variable, and two-way ANOVA for datasets with three or more groups involving two variables. All analyses were conducted with GraphPad Prism 9 software.

## 3. Results

### 3.1. Drp1 Is Phosphorylated upon BCR Stimulation and Is Recruited with Mitochondria and Lysosomes to the Immune Synapse

**Figure 1:**
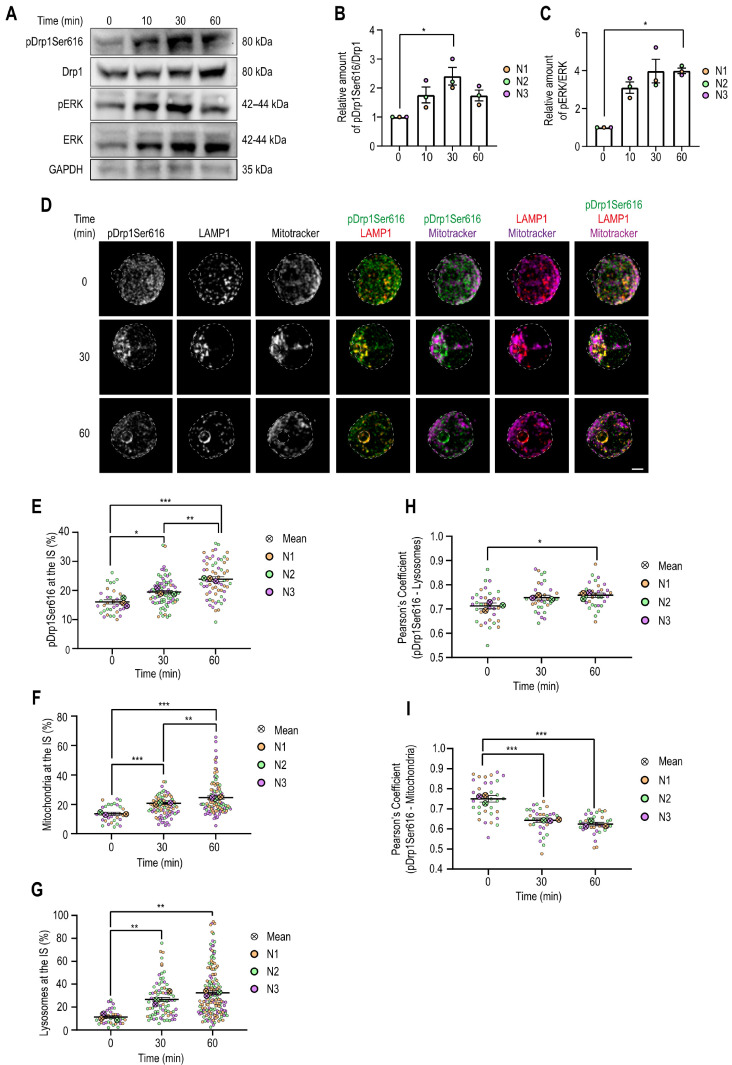
Drp1 is phosphorylated in response to BCR activation and is recruited to the B cell IS. (**A**) Immunoblot of B cells activated on BCR ligand+-coated plates for indicated times, where phosphorylated and total levels of DRP and ERK were detected. Quantification of relative amounts of (**B**) pDrp1Ser616 and (**C**) pERK, representative of three independent experiments. (**D**) Representative confocal planes of B cells activated with antigen-coated beads, stained for pDrp1Ser616 (Green); LAMP1: lysosome-associated protein 1 (red); and Mitotracker DeepRed (magenta). Dashed lines indicate bead position and cell boundaries, respectively. Scale bar: 3 µm. Quantification of (**E**) pDrp1Ser616, (**F**) mitochondria and (**G**) lysosomes associated with B cell IS. Quantification of colocalization index via whole-cell Pearson’s coefficient between (**H**) pDrp1Ser616 and lysosomes or (**I**) pDrp1Ser616 and mitochondria. Data information: In (**B**,**C**,**E**–**I**), data are presented as independent experiments; the mean represents the average between each independent mean. N = 3, n > 30 in each condition. *, **, *** = significant differences (* *p* < 0.05; ** *p* < 0.01; *** *p* < 0.001; one-way ANOVA, Tukey’s multiple comparisons test).

**Figure A1:**
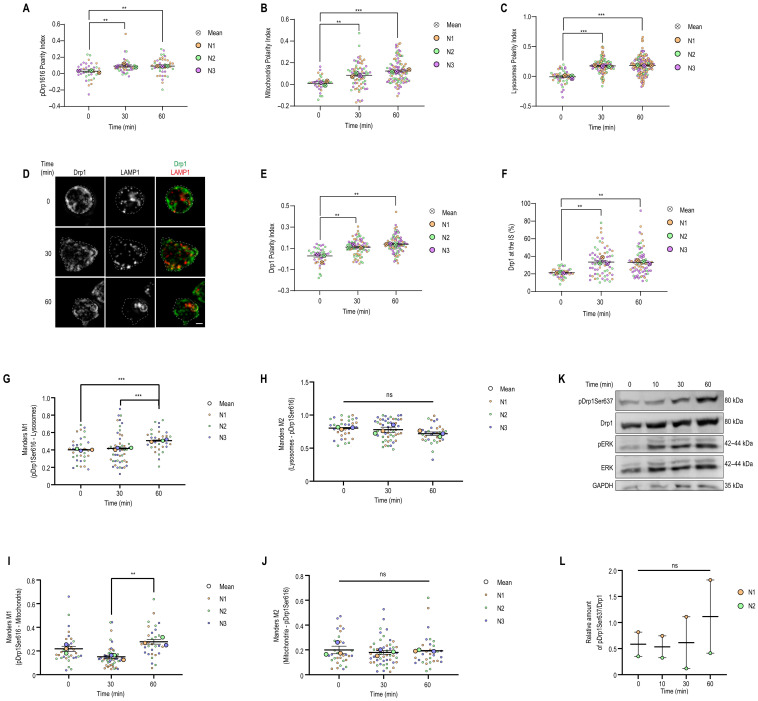
Phosphorylated Drp1, mitochondria and lysosomes are recruited to the IS on B cells. Quantification of polarity index of (**A**) pDrp1Ser616, (**B**) mitochondria and (**C**) lysosomes to the IS on B cells. N = 3, n > 30 in each experiment. **, *** = significant differences (** *p* < 0.01; *** *p* < 0.001). (**D**) Representative images of activated B cells with beads coated with BCR+ ligand. Each image corresponds to the standard deviation of the fluorescence intensity of the planes obtained via epifluorescence microscopy. Drp1 (Green), LAMP1: lysosome-associated protein 1 (red). Dashed lines indicate bead position and cell boundaries, respectively. Scale bar: 3 µm. Quantification of Drp1 (**E**) polarity index and (**F**) associated with the IS of B cells. N = 3, n > 30 in each experiment. **, *** = significant differences (** *p* < 0.01; *** *p* < 0.001); ns = non-significant differences (*p* > 0.05). Manders’ Colocalization Coefficients from (**G**) M1 pDrp1Ser616 in Lysosomes, (**H**) M2 Lysosomes in pDrp1Ser616, (**I**) M1 pDrp1Ser616 in Mitochondria and (**J**) M2 Mitochondria in pDrp1Ser616. (**K**) Immunoblot of B cells activated on BCR ligand+-coated plates for indicated times, where phosphorylated and total levels of DRP and ERK were detected. (**L**) Quantification of relative amounts of pDrp1Ser637.

To determine whether B cell immune synapse formation is associated with mitochondrial dynamics, we evaluated the activation of Drp1, a GTPase that exerts mitochondrial fission upon phosphorylation at its Ser616 residue [24,25]. To this end, a B lymphoma cell line was plated onto antigen-coated surfaces containing BCR ligands for defined time intervals to mimic B cell activation and immune synapse formation. Next, cells were lysed and analyzed by immunoblot. Our results show that activated B cells displayed higher levels of phosphorylated Drp1 (pDrp1-Ser616) (Figure 1A,B), suggesting B cell activation is coupled with changes in mitochondria dynamics (Figure 1C). To examine mitochondrial organization during B cell activation, we used antigen-coated beads, an established model that mimics B cell engagement with immobilized antigen and induces the formation of a polarized immune synapse [4,6]. Cells were labeled with MitoTracker and stained for pDrp1-Ser616 and the lysosomal marker LAMP1. Both pDrp1-Ser616 and mitochondria were recruited to the immune synapse (Figure 1D), as confirmed by polarity index measurements (Figure A1A,B) and quantification of their association with the synaptic site (Figure 1E,F). Total Drp1 was also recruited to the synaptic membrane under these conditions (Figure A1D–F). Consistent with previous reports, lysosomes also accumulated at the IS in close proximity to the antigen-coated bead (Figure 1D,G and Figure A1C), indicating coordinated recruitment with activated Drp1 and mitochondria. Interestingly, pDrp1-Ser616 localized spatial proximity to LAMP1+ vesicles after activation, suggesting a possible association between activated Drp1 and lysosomes. Consistent with this observation, Pearson’s correlation analysis revealed a progressive increase in colocalization between pDrp1-Ser616 and LAMP1 over time (Figure 1H), whereas colocalization between pDrp1-Ser616 and mitochondria remained unchanged (Figure 1I). Similar results were obtained using Manders’ colocalization coefficients. Specifically, the fraction of pDrp1-Ser616 signal overlapping with lysosomes (Manders’ M1) increased over time (Figure A1G), while the fraction of lysosomal signal overlapping with pDrp1-Ser616 (Manders’ M2) remained unchanged (Figure A1H). In contrast, analysis of pDrp1-Ser616 and mitochondria showed an increase in the fraction of pDrp1-Ser616 signal overlapping with mitochondria (M1, Figure A1I), whereas the fraction of mitochondrial signal overlapping with pDrp1-Ser616 remained stable (M2, Figure A1J). Since Drp1 can also be phosphorylated at Ser637, a modification associated with inhibitory activity [25,26], we evaluated this form in activated B cells. Our results showed that pDrp1-Ser637 levels remained unchanged throughout activation (Figure A1K,L). These findings suggest that BCR stimulation promotes the redistribution of activated Drp1 toward lysosome-enriched regions at the immune synapse, where it may contribute to the coordination of organelle dynamics during B cell activation.

### 3.2. Mdivi-1 Treatment Is Associated with Reduced Drp1 Activation and Decreased Recruitment of Mitochondria to the Immune Synapse of B Cells

**Figure 2:**
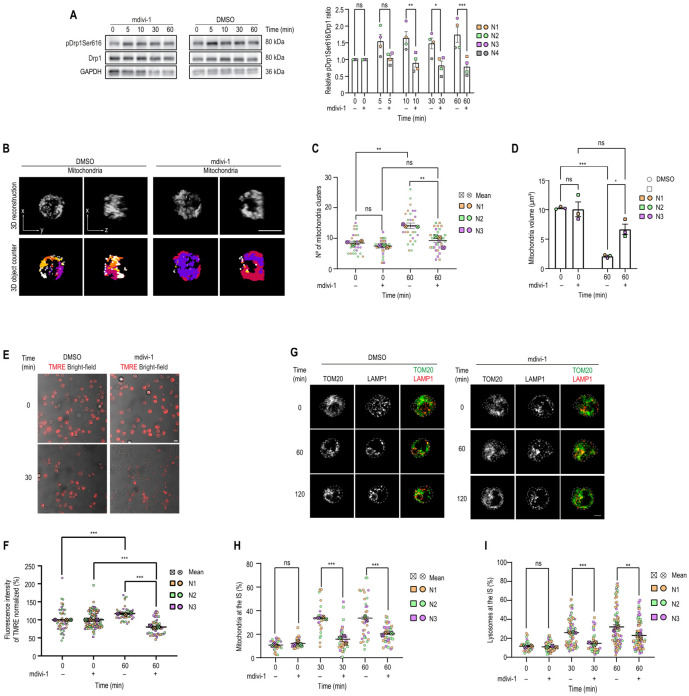
Mdivi-1 treatment is associated with reduced Drp1 phosphorylation and decreased recruitment of mitochondria and lysosomes to the IS of B cells. (**A**) Detection of pDrp1Ser616 in B cells treated with 50 µM mdivi-1 or DMSO and activated on BCR ligand+-coated plates for indicated times. Right: Quantification of relative amounts of pDrp1Ser616 Representative of three independent experiments. (**B**) (Top) scheme of the 3D reconstruction of the images obtained by confocal microscopy from activated B cells treated with DMSO or mdivi-1. (Bottom) Representation of the identification of individual objects for the quantification of cluster numbers and mitochondrial cluster volume from activated B cells treated with DMSO or mdivi-1. Scale bar: 10 µm. From the images acquired by confocal microscopy, each cell was isolated from the field of view and independently reconstructed in 3D using ImageJ. The ImageJ 3D object counter plugin was used to quantify independent clusters and cluster volume. Quantification of (**C**) number of clusters and (**D**) volume of mitochondria in B cells treated with 50 µM mdivi-1 or DMSO and activated for 0 or 60 min. (**E**) B cells treated with 50 µM mdivi-1 or DMSO and activated for 0 or 30 min were stained with TMRE dye to label active mitochondria. Scale bar: 10 µm. (**F**) Quantification of fluorescent intensity from TMRE dye in DMSO and mdivi-1-treated cells. (**G**) Representative confocal images of B cells treated with 50 µM mdivi-1 or DMSO for 4 h and activated with antigen-coated beads for indicated time points. Cells were fixed and stained for TOM20 (green), LAMP1 (red). Dashed lines indicate bead position and cell boundaries, respectively. Scale bar: 3 µm. Quantification of (**H**) mitochondria and (**I**) lysosomes associated with IS. Data information: In (**A**,**C**,**D**,**F**,**H**,**I**) data are presented as independent experiments; the mean represents the average between each independent mean. N = 3, n > 30 in each condition. *, **, *** = significant differences (* *p* < 0.05; ** *p* < 0.01; *** *p* < 0.001; one-way ANOVA, Tukey’s multiple comparisons test); ns = non-significant differences (*p* > 0.05, one-way ANOVA, Tukey’s multiple comparisons test).

To examine whether mitochondrial remodeling contributes to mitochondrial recruitment during B cell immune synapse formation, we used mdivi-1, a pharmacological compound widely employed to perturb Drp1-associated mitochondrial fission and mitochondrial activity [20]. To assess the effect of mdivi-1 on Drp1 phosphorylation, B cells were pretreated for 4 h with 50 µM mdivi-1 or DMSO and subsequently activated on antigen-coated surfaces for different time points. Immunoblot analysis of B cell lysates showed reduced pDrp1-Ser616 levels in mdivi-1-treated cells relative to controls, consistent with reduced activation of Drp1 (Figure 2A). We next evaluated the effects of mdivi-1 treatment on the activation of B cells and focused on changes in the number and/or volume of mitochondria, which are regulated by Drp1. For this purpose, the images acquired by confocal microscopy were reconstructed in 3D images, and the analysis of mitochondria from non-activated and activated B cells was performed (Figure 2B). In BCR-stimulated control cells, we observed an increase in the number of mitochondrial clusters compared to non-activated cells, revealing changes in mitochondrial dynamics associated with B cell activation. In contrast, mdivi-1-treated B cells did not display changes in cluster number upon activation (Figure 2C). Moreover, mitochondrial volume analysis revealed that B cell activation induced a significant reduction in mitochondrial volume under control conditions (Figure 2D), and this effect was significantly attenuated in mdivi-1-treated B cells. These findings suggest that B cell activation promotes mitochondrial remodeling with features compatible with increased Drp1-linked fission, a process that is sensitive to mdivi-1 treatment. We next assessed mitochondrial function during B cell immune synapse formation. To this end, we used TMRE, a fluorescent dye that accumulates in the mitochondrial matrix of active mitochondria [27]. Our results show that upon activation mitochondrial activity increased in control cells, which was significantly lower in mdivi-1-treated B cells after 30 min of activation (Figure 2E,F). These results suggest that mdivi-1 treatment limits the increase in mitochondrial activity normally observed during B cell activation, consistent with altered mitochondrial remodeling and/or mitochondrial respiratory activity under these conditions.

**Figure A2:**
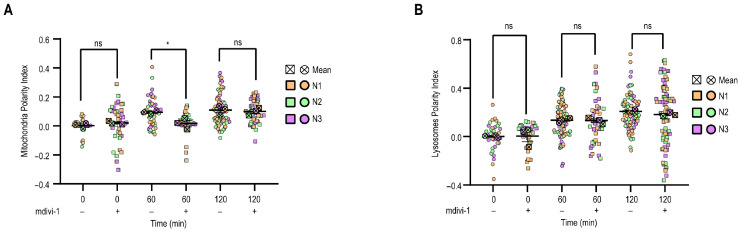
Recruitment of mitochondria to the IS decreased in mdivi-1-treated B cells. Quantification of polarity index of (**A**) mitochondria and (**B**) lysosomes to the IS of B cells. B cells treated with DMSO or mdivi-1 50 µM for 4 h were activated for 0, 60 and 120 min N = 3, n > 30 in each experiment. * = significant differences (* *p* < 0.05); ns = non-significant differences (*p* > 0.05).

We next asked whether mdivi-1 treatment affects the recruitment of mitochondria and lysosomes to the IS during B cell activation. To this end, B cells were preincubated for 4 h with 50 µM mdivi-1 or DMSO as a control, and then activated with beads coated with BCR ligands for the indicated times. Cells were subsequently stained for the mitochondrial marker TOM20 and the lysosomal marker LAMP1 (Figure 2G). We found that mdivi-1-treated B cells showed a reduced percentage of both mitochondria and lysosomes associated with the IS compared with control cells (Figure 2H,I). However, analysis of the polarity index revealed no significant differences in the positioning of either organelle relative to the IS (Figure A2A,B), suggesting that mdivi-1 affects their stable association with the synaptic membrane rather than their overall polarization or transport. Altogether, these results indicate that mdivi-1-sensitive changes in mitochondrial remodeling and activity are associated with reduced recruitment of mitochondria and lysosomes to the IS.

### 3.3. Mdivi-1 Treatment Enhances Tubulin Acetylation of B Cells

**Figure 3:**
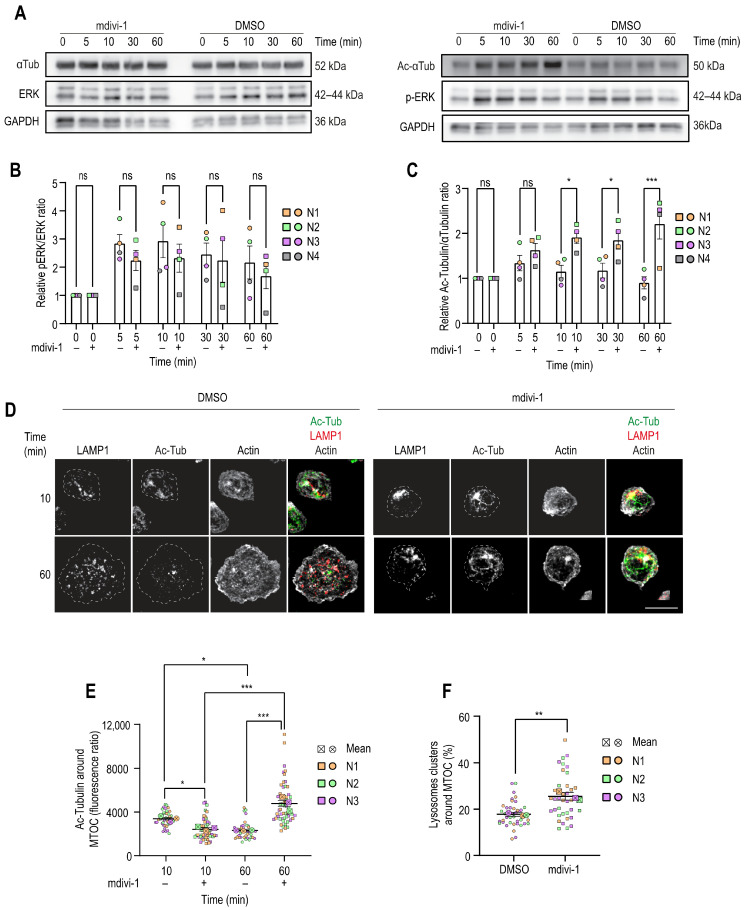
Increased acetylated tubulin around the MTOC after mdivi-1 treatment in B cells. (**A**) Detection of pERK and acetylated microtubules (Ac-α-Tub) in B cells treated with 50 µM mdivi-1 or DMSO and activated on BCR ligand+ coated plates for the indicated times. (**B**,**C**) Quantification of relative amounts of pERK and Ac-α-Tub, respectively. N = 4. *, *** = significant differences (* *p* < 0.05; *** *p* < 0.001); ns = non-significant differences (*p* > 0.05). (**D**) Representative confocal images of B cells treated with 50 µM mdivi-1 or DMSO for 4 h and activated with antigen-coated slides for indicated time points. Cells were fixed and stained for acetylated tubulin (green), LAMP1 (red), Actin (greyscale). Dashed lines indicate bead position and cell boundaries, respectively. Scale bar: 10 µm. (**E**) Quantification of the amount of acetylated α-tubulin associated with MTOC in B cells activated for 60 min. N = 3, n > 30 in each experiment. *, **, *** = significant differences (* *p* < 0.05; ** *p* < 0.01; *** *p* < 0.001), ns = non-significant differences (*p* > 0.05). (**F**) Quantification of lysosome clusters around MTOC from B cells treated with DMSO or 50 µM mdivi and activated for 60 min. N = 3, n > 30 in each experiment. *, *** = significant differences (* *p* < 0.05, *** *p* < 0.001); ns = non-significant differences (*p* > 0.05).

Because BCR activation is initiated by antigen binding at the surface of antigen-presenting cells [1,2,3], we next examined whether mdivi-1 affects downstream BCR signaling in activated B cells. To this end, B cells treated with mdivi-1 or DMSO were stimulated on surfaces coated with BCR ligands for the indicated times. Cell lysates were then analyzed by Western blot to assess levels of BCR downstream signaling proteins, including ERK and phospho-ERK [28]. We did not detect significant differences in p-ERK/ERK levels between activated B cells treated with DMSO and those treated with mdivi-1 (Figure 3A,C). These results suggest that, under conditions in which mdivi-1 alters mitochondrial remodeling and activity, proximal downstream BCR signaling is not substantially impaired.

To gain mechanistic insight into how mdivi-1 affects lysosome association and mitochondrial recruitment to the immune synapse, we next examined the post-translational modifications of microtubules, particularly acetylation, a marker associated with microtubule stability [29] that was recently shown to regulate lysosome positioning at the immune synapse [30]. Additionally, mitochondria are often enriched in regions containing acetylated microtubules [31,32], and they can also contribute to microtubule acetylation through the MFN2-dependent recruitment of ATAT1, thereby influencing their own mobility along the microtubule network [33]. Based on this, we asked whether acetylated tubulin dynamics were altered in our model. To address this, we measured acetylated α-tubulin levels relative to total α-tubulin in activated B cells and evaluated the effect of mdivi-1 treatment by immunoblot. Consistent with previous reports, α-tubulin acetylation increased upon B cell activation [5]. Notably, acetylated α-tubulin levels were significantly higher in mdivi-1-treated activated B cells than in control cells (Figure 3A,C), in agreement with findings in other systems [33]. These results suggest that increased α-tubulin acetylation may promote lysosome accumulation around the MTOC, where these organelles could become stabilized.

**Figure A3:**
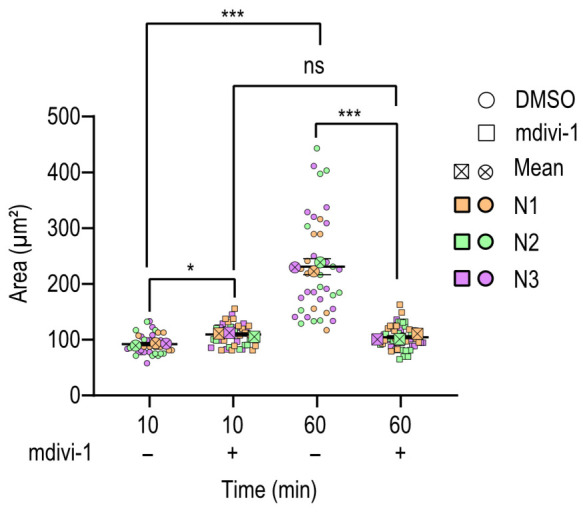
B cell spreading responses decrease upon treatment with mdivi-1. Quantification of the spreading area of B cells activated on BCR^+^ ligand-coated slides and stained for F-actin. N = 3, n > 30 in experiment. N = 3, n > 30 in each experiment. *, ***= significant differences (*p < 0.05 *** *p* < 0.001); ns = non-significant differences (*p* > 0.05).

We further investigated the effect of mdivi-1 on microtubule acetylation and its impact on lysosome distribution during immune synapse formation. To this end, B cells were treated with mdivi-1 or DMSO and activated on antigen-coated surfaces for the indicated times, allowing us to evaluate synaptic membrane remodeling and lysosome organization. Under these conditions, mdivi-1-treated B cells showed reduced spreading responses (Figure A3), indicating a diminished capacity to respond to immobilized antigen. Moreover, imaging analysis revealed increased levels of acetylated α-tubulin in mdivi-1-treated cells (Figure 3D,E), in agreement with Western blot analysis. Acetylated microtubules were also closely associated with lysosome clusters (Figure 3D,F), suggesting that enhanced microtubule acetylation may retain lysosomes in the pericentrosomal region near the MTOC and thereby restrict their fusion at the immune synapse.

### 3.4. Mdivi-1 Treatment Affects Lysosome Distribution and Fusion at the B Cell Synaptic Interface

**Figure 4:**
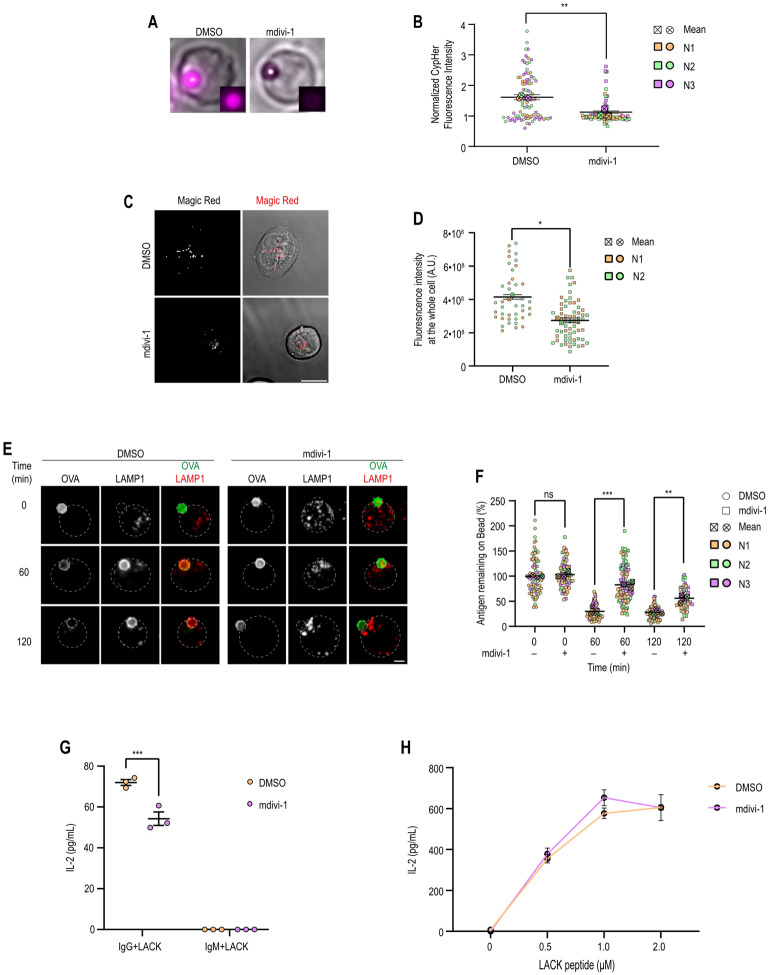
Reduced lysosome secretion and antigen extraction at the immune synapse of B cells upon treatment with mdivi-1. (**A**) Representative epifluorescence images of B cells pretreated with DMSO or 50 µM mdivi-1 for 4 h and activated with CypHer5E-coupled BCR ligand+ beads for 90 min. Cells are shown as sum of projections of a stack. Insets highlight the CypHer5E fluorescence in the bead. Scale bar: 3 μm. (**B**,**F**) Quantification of the bead CypHer5E intensity, normalized by the intensity of noninteracting beads and activated for 90 min with beads coated with CypHer 5E-coupled BCR cognate antigen and plated on poly-L-lysine-coated coverslips. (**C**) Representative confocal images of B cells pretreated with DMSO or 50 µM mdivi-1 for 4 h and activated on BCR ligand+ slides for 30 min and stained with magic-red. The images correspond to the sum of the acquired planes. (**D**) Quantification of the fluorescence of magic-red on B cells. N = 2, n ≥ 20 in each experiment. * = significant differences (* *p* < 0.05); ns = non-significant differences (*p* > 0.05). (**E**) Immunofluorescence of B cells treated with DMSO (left) or 50 µM mdivi-1 (right) for 4 h and activated with BCR ligand+ beads containing OVA. Cells were fixed and stained for OVA (green) and LAMP1 (red). Dashed lines indicate bead position and cell boundaries, respectively. Scale bar: 3 µm. Each image corresponds to the sum of the acquired planes through epifluorescence microscopy. (**F**) Quantification of the amount of OVA remaining on the bead after each activation time. N = 3, n > 30 in each experiment. **, *** = significant differences (** *p* < 0.01; *** *p* < 0.001); ns = non-significant differences (*p* > 0.05). (**G**) Antigen presentation assay of B cells treated with DMSO or 50 µM mdivi-1 for 4 h. Cells were incubated with activating (BCR^+^ ligand) or non-activating (BCR^−^ ligand) beads coupled to Lack protein. Next, cells were fixed, washed and co-cultured for 4 h with T cells. Quantification of IL-2 levels produced by T cells; (**H**) peptide control of the presentation assay. N = 3, n > 30 in each experiment. *, **, *** = significant differences (* *p* < 0.05; ** *p* < 0.01; *** *p* < 0.001); ns = nonsignificant differences (*p* > 0.05).

To determine whether enhanced lysosome accumulation at the centrosome region translated into impaired lysosome secretion at the synaptic membrane, we incubated control and mdivi-1-treated B cells with beads containing BCR ligand+ coupled to CypHer5E, a dye whose fluorescence increases at acidic pH [4]. Consistent with the results above, we observed reduced fluorescence in beads interacting with mdivi-1-treated B cells (Figure 4A,B), indicating the reduced fusion capacity of lysosomes in these cells.

As previously reported, impaired polarized lysosome secretion at the IS of B cells dramatically affects their capacity to extract and present immobilized antigens in vitro [4,5]. We therefore investigated whether analogous defects were observed in mdivi-1-treated cells. We first monitored the lysosome hydrolase activity in B cells that were treated or not with mdivi-1. To this end, B cells were treated with mdivi-1, activated for 30 min on BCR+ ligand-coated surfaces and stained with magic-red (MR), which penetrates cells and fluoresces upon cleavage within catalytically active lysosomes [34]. Imaging analysis revealed lower MR+ fluorescence in mdivi-1-treated B cells (Figure 4C,D), indicating lower lysosomal cathepsin activity. We next evaluated whether such lysosome defects translate into lower antigen extraction and presentation. For this purpose, we assessed antigenic extraction by measuring the fluorescence signal of ovalbumin (OVA) remaining in beads interacting with B cells under different conditions. B cells were treated with mdivi-1 or DMSO and subsequently activated with beads coated with BCR+-ligand plus OVA. We observed that mdivi-1-treated B cells show a higher number of OVA remaining on the beads compared to DMSO-treated B cells with the same activation time (Figure 4E,F).

**Figure A4:**
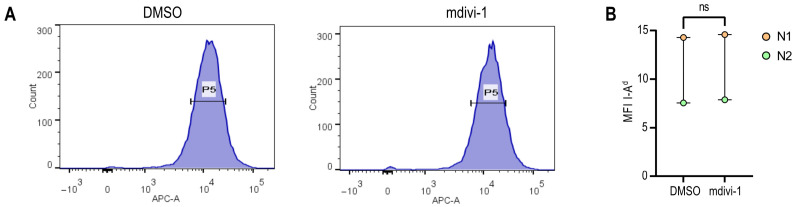
Treatment with mdivi-1 does not alter the cell surface levels of MHC class II in B cells. (**A**) Representative histogram for I-A^d^ from B cells treat with (Left) DMSO or (Right) mdivi-1. (**B**) MFI from I-A^d^ on B cells treated with DMSO or mdivi-1 for 4 h. (Right). ns = non-significant differences (*p* > 0.05).

To identify the impact on antigen presentation, we treated B cells with mdivi-1 or DMSO and incubated them with beads coupled to BCR+-ligand, together with Lack protein from Leishmania major, as previously described [5]. Subsequently, B cells were co-cultured with a specific T cell hybridoma and their ability to present MHC-II-peptide complexes, derived from Lack protein-associated beads, was measured through the levels of IL-2 secreted by activated cells. In this case, we observed lower IL-2 production in T cells co-cultured with mdivi-1-treated B cells compared to co-cultures of T cells with DMSO-treated B cells (Figure 4G). No difference in IL-2 levels produced by T cells co-cultured with mdivi-1 or DMSO-treated B cells was observed in the peptide control assay (Figure 4H), suggesting no effect on MHC-II cell surface levels were caused by mdivi-1. Consistent with this interpretation, surface levels of I-Ad were comparable between DMSO- and mdivi-1-treated B cells (Figure A4). Together, these results indicate that mdivi-1-sensitive changes in mitochondrial dynamics and activity are associated with reduced lysosome function, antigen extraction, and antigen presentation.

## 4. Discussion

In this study, we identify mitochondrial remodeling as an early component of B cell immune synapse formation and function. Although mitochondria are well recognized as supporting the metabolic demands of activated B cells [10,13], their direct contribution to antigen extraction and presentation at the IS has remained unclear. Our data show that BCR engagement by immobilized antigen is accompanied by the rapid phosphorylation of Drp1 at Ser616, changes in mitochondrial morphology, and mitochondrial accumulation at the synaptic interface. Pharmacological perturbation with mdivi-1 reduced Drp1 phosphorylation, impaired mitochondrial remodeling and synaptic accumulation, and was associated with defective lysosome positioning, lysosome fusion, antigen extraction, and antigen presentation. Together, these findings support a model in which Drp1-associated mitochondrial dynamics contribute to the organization and function of the B cell IS.

In this study, mdivi-1-treated B cells showed reduced mitochondrial metabolic activity. However, because mdivi-1 has also been reported to act as a reversible inhibitor of mitochondrial complex I of the electron transport chain, we cannot exclude the possibility that this effect reflects impaired electron transport rather than selective Drp1 inhibition alone. This off-target effect could involve ROS-sensitive pathways and may also influence AMPK signaling or ERK-dependent regulation downstream of BCR activation [35,36]. Additional approaches, such as Seahorse oxygen consumption rate (OCR) measurements or ATP/ADP ratio analysis, will therefore be required to directly assess mitochondrial metabolism. Moreover, in other cellular models, mdivi-1 has been shown to reduce Drp1 phosphorylation at Ser616 by interfering with Drp1 activation [37]. Thus, although our results suggest that Drp1 inhibition is associated with altered mitochondrial function in activated B cells, genetic approaches such as siRNA- or shRNA-mediated Drp1 silencing will be necessary to validate this conclusion.

These results extend previous studies showing that B cell activation is coupled to metabolic reprogramming and mitochondrial remodeling. Activated B cells increase glycolysis and oxidative phosphorylation and undergo changes in mitochondrial morphology that accompany differentiation and antibody responses [13,38]. Our findings suggest that mitochondrial remodeling is not only a later metabolic adaptation, but also an early and spatially coordinated event during IS formation. The increase in mitochondrial cluster number and the reduction in mitochondrial volume observed upon activation are consistent with enhanced mitochondrial fission, supporting the idea that BCR engagement rapidly reconfigures the mitochondrial network to meet the local demands of synapse formation [23].

A key observation of this study is that Drp1 becomes phosphorylated during B cell activation and accumulates together with mitochondria at the IS. Phosphorylation of Drp1 at Ser616 has been associated with mitochondrial fission in multiple systems [18,19,20], and our results are consistent with the activation of this pathway following BCR stimulation. Moreover, pDrp1-Ser616 increasingly associated with LAMP1-positive compartments over time, whereas its colocalization with mitochondria decreased after activation. Although this does not demonstrate a direct interaction, it suggests that activated Drp1 redistributes toward lysosome-rich regions at the synapse, where it may contribute to the coordination of mitochondrial and lysosomal dynamics. This interpretation is consistent with studies in T cells showing that Drp1-associated mitochondrial fission supports mitochondrial recruitment to the IS and sustains the localized signaling required for activation [20,39].

Our data further indicate that mitochondrial dynamics are functionally linked to lysosome behavior in activated B cells. mdivi-1 treatment reduced the association of both mitochondria and lysosomes with the synaptic membrane, whereas polarity index measurements were less affected, suggesting that organelles remain polarized toward the contact site but fail to establish stable association with the IS. This distinction suggests that mitochondrial remodeling may be particularly important for the final positioning of these organelles at the synaptic interface rather than for their initial polarization. Consistent with this idea, the perturbation of mitochondrial fission by mdivi-1 was associated with multiple signs of lysosome dysfunction, including reduced lysosome fusion at the synaptic membrane, decreased cathepsin activity, impaired extraction and the presentation of immobilized antigen.

Mechanistically, our results also suggest a link between mitochondrial dynamics and microtubule organization. mdivi-1 treatment increased acetylated α-tubulin levels and promoted the accumulation of lysosome clusters in association with acetylated microtubules near the MTOC. Because microtubule acetylation is associated with increased stability, one possible interpretation is that sustained acetylation favors lysosome retention in the pericentrosomal region, thereby limiting their delivery to the synaptic membrane [26,40]. These observations raise the possibility that Drp1-associated mitochondrial remodeling occurs in coordination with lysosome dynamics and may contribute indirectly to lysosome secretion by supporting a microtubule state that is permissive for lysosome release and fusion at the IS. However, specific targeting of Drp1 will be required to define the molecular basis of this connection.

Our findings are also relevant considering recent evidence showing that defective mitochondrial remodeling in B cells alters lysosomal organization and promotes features of an aged humoral response [21]. That study established a connection between mitochondria and lysosomal remodeling in B cells, but the underlying mechanisms remained unresolved. The present work refines this relationship by focusing on the early events triggered by BCR engagement with immobilized antigen and identifying Drp1-associated remodeling as a candidate mechanism linking mitochondrial dynamics to lysosome positioning and function at the IS.

In summary, our results support a model in which BCR engagement with immobilized antigen is accompanied by Drp1 phosphorylation, mitochondrial accumulation at the IS, and changes in mitochondrial dynamics and activity that are sensitive to mdivi-1. Under these conditions, mdivi-1 treatment is associated with increased microtubule acetylation, altered lysosome positioning and secretion, and reduced antigen extraction and presentation by B cells. These findings suggest that mitochondrial dynamics contribute to immune synapse organization and function beyond their established role in metabolic support.

## Data Availability

The original contributions presented in this study are included in the article. Further inquiries can be directed to the corresponding author.
